# Coming home: how visually navigating ants (*Myrmecia* spp.) pinpoint their nest

**DOI:** 10.1242/jeb.249499

**Published:** 2025-01-27

**Authors:** Jochen Zeil

**Affiliations:** Research School of Biology, The Australian National University, 46 Sullivans Creek Road, Canberra ACT2601, Australia

**Keywords:** Insects, Ants, Homing, Visual navigation

## Abstract

Visually navigating *Myrmecia* foragers approach their nest from distances up to 25 m along well-directed paths, even from locations they have never been before ( Narendra et al., 2013). However, close to the nest, they often spend some time pinpointing the nest entrance, sometimes missing it by centimetres. Here, I investigated what guides homing ants in their attempt to pinpoint the nest entrance. As the ants approach the nest, their behaviour changes. At approximately 1 m from the nest, the ants slow down, their scanning amplitude becomes larger and their path direction changes more frequently. This change in scanning behaviour is not triggered by local olfactory, tactile or visual cues because ants tethered on a trackball 30–50 cm above ground also exhibit it at 0.6 m compared with 1.6 m distance from the nest*.* Moreover, the ants are able to pinpoint the nest when such local cues are removed by covering the ground around the nest or the nest entrance itself. *Myrmecia* ants thus rely on information from the global panorama when pinpointing the nest. During learning walks, these ants appear to systematically collect views directed toward and away from the nest ( Jayatilaka et al., 2018). Homing ants indeed change gaze and body axis direction appropriately with a delay when encountering views to the left or to the right of the nest. However, image analysis shows that close to the nest, opponent views with the same orientation become too similar, explaining the growing uncertainty reflected in the ants' increased scanning behaviour during homing.

## Teaser

**Summary:** Visually navigating Australian bull ants find their way back to their nest from large distances, but often have difficulties with pinpointing the entrance. Detailed analysis of their behaviour reveals why and how they succeed.

## INTRODUCTION

In the open woodland habitats of *Myrmecia croslandi* ants, nest-directed panoramic views that the ants experience and presumably memorize during their learning walks (Jayatilaka et al., 2018) can provide navigational guidance over distances of tens of metres, if they are compared with what an ant currently sees (Narendra et al., 2013). Within the catchment area of memorized nest-directed views, the minimum of the rotational image difference function (rotIDF) between such views and the view currently seen points into the direction of the nest (Graham et al., 2010; Narendra et al., 2013; Stürzl et al., 2015). As an ant approaches the nest location, the depth of the rotIDF becomes larger (its minimum becomes smaller) because the image differences due to translation (transIDF) between learnt views and current views become smaller (Narendra et al., 2013; Stürzl et al., 2015; Zeil, 2023). Indeed, homing simulations using reconstructed views from a *M. croslandi* foraging environment bring an agent to within 1 m of a goal location (Le Moël and Wystrach, 2020).

However, close to the nest, this global guidance becomes less and less reliable, because the differences between closely spaced panoramic views with the same orientation become very small (e.g. Jayatilaka et al., 2018; Murray et al. 2020). Yet, successful homing requires centimetre accuracy and so far it remains unclear – at least for visually navigating *Myrmecia* ants – what cues are involved in a returning ant's final approach to the nest. Desert ants, *Cataglyphis fortis*, for instance, are guided by tactile and olfactory cues when pinpointing their visually inconspicuous nest entrance in the visually featureless salt-pan landscape (Seidl and Wehner, 2006; Buehlmann et al., 2012, 2020).

Here, I provide a detailed analysis of the changes of behaviour shown by individually foraging, day-active Australian jack-jumper ants when they approach the nest, and show that even very close to the nest entrance they are guided by scene memories which they must have acquired during their learning walks.

## MATERIALS AND METHODS

I observed, and performed experiments with, foragers of two identified (*Myrmecia croslandi* and *Myrmecia impaternata*) and one unidentified species (*Myrmecia* sp.) of day-active Australian jack-jumpers of the *Myrmecia pilosula* species complex (Taylor, 2015).

**Fig. 1.**
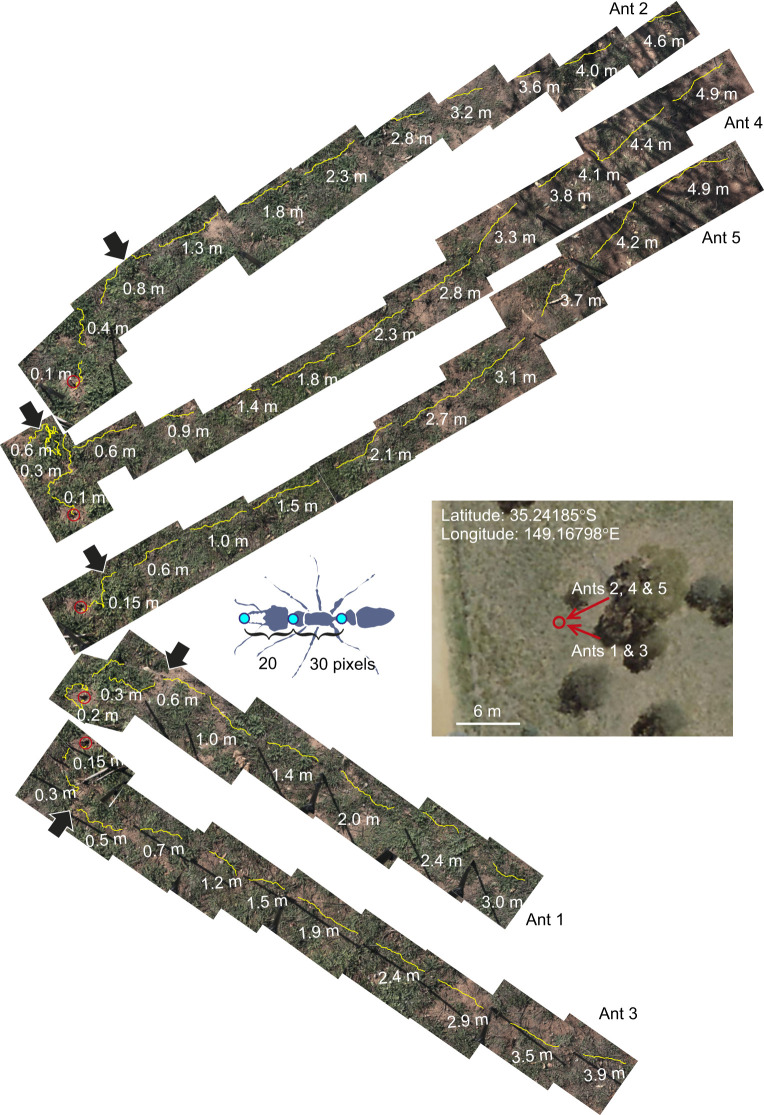
**The homing paths of five *Myrmecia* sp. foragers.** Paths were filmed with a 4K camcorder on a tripod, which was moved whenever an ant was about to exit the field of view. The *x*/*y* coordinates of mandible tips, the back of the head and the petiole were digitized for each stationary camera sequence (as indicated in the inset) and the head coordinate plotted over each of the consecutive scenes in yellow. Using common objects in subsequent scenes, the images were aligned by eye to show the whole paths. Gaps in the paths are due to camera shifts. Numbers indicate distance to the nest (labelled by a red circle) measured halfway along each path segment. The orientation of the paths is roughly aligned with their real-world directions as indicated by red lines in the aerial image of the nest site. Arrows point to large, sustained changes in heading direction.

Five *Myrmecia* sp. foragers from a nest at the lower slopes of Mount Majura Nature Reserve in the Australian Capital Territory (35°14.50290′S, 149°10.09474′E) were filmed on 28 April 2020 returning to the nest between 12:00 and 16:00 h local time. Upon detecting an ant, a tripod-mounted Sony FDR-AX100E 4K Camcorder was positioned over her and moved without changing the camera orientation and height whenever the ant was about to leave the recording area until she had reached the nest. The distances from the nest at which an ant was first detected ranged from 3 to 4.9 m. The frame rate was 25 frames s^−1^, and at a 3840×2160 pixel image size and a recording area of 0.75×0.42 m, the resolution was sufficient to subsequently track frame-by-frame the tip of mandibles and the back of the head (∼20 pixel length; Fig. 1 inset) and her pronotum (back of head to pronotum, ∼30 pixel length), using a custom-written MATLAB program (MathWorks, Natick, MA, USA) (digilite, Robert Parker and Jan Hemmi, The Australian National University). From this, the horizontal orientation of the head and of the longitudinal body axis could be determined to within at least 10 deg. Still images of successive camera positions were later stitched by hand to align path segments and to determine the mean distance of each segment to the nest (Fig. 1). *x*/*y* positions and angular velocities [e.g. change in gaze direction, 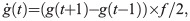 where *ġ* is the change in gaze direction (deg s^−1^), *g* is gaze direction (deg) and *f* is frame rate (frames s^−1^); and speed, 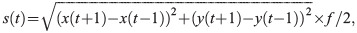 where *s* is speed (cm s^−1^), and *x* and *y* are positions of the body centre (cm)] were smoothed with a three-point averaging filter.

### Statistics

Medians of gaze direction, body axis orientation, head direction relative to the body, path direction and speed of the five ants that were tracked to the nest from distances between 3 and 4.9 m away were sorted into 0.5 m distance bins. To provide a balanced data set, the minimum number of samples across all distance bins was determined to be 100 and for each ant, 100 samples were selected randomly from distance bins with more than 100 samples. A two-sided Wilcoxon rank sum test was used (using the MATLAB ranksum function) to test the null hypothesis that variables at distances smaller than 1 m from the nest and the same number of samples for distances between 2 and 3 m from the nest (distance bins to which all five ants contributed) have equal medians. Analysis was carried out in MATLAB 2022b.

Other paths and gaze directions of returning foragers close to the nest were filmed on 10 March 2020 between 11:00 and 12:00 h (20 paths), and on 5 March 2021 (>30 paths) between 09:00 and 11:00 h with a stationary Sony FDR-AX100E 4K camcorder (recording area 1.05×0.59 m) at a *M. croslandi* nest on the campus of the Australian National University (ANU; 35°17.35285′S, 149°6.95037′E) and analysed in the same way.

In addition, three *M. croslandi* ants were tethered over an air-cushioned trackball (for details, see Murray et al., 2020) that was placed on a camera dolly that could be moved along a rail track from 1.6 to 0.6 m away from the ANU nest. The ants were approximately 50 cm above ground and were free to rotate on the trackball. They were filmed from above with an iPhone 6 at 120 frames s^−1^ and an image size of 1920×1080 pixels. Their gaze directions (head orientation) were determined frame-by-frame for 20 s each, when the camera dolly was stationary at 1.6 m and when it was 0.6 m away from the nest, respectively. This experiment was repeated in 2024 at a nest of *M. impaternata* in Mount Majura Nature Reserve in the Australian Capital Territory (35°14.683′S, 149°10.282′E) with 17 ant foragers that were caught close to the nest entrance as they returned from different compass directions. The ants thus ceased to receive directional information from their path integration system (zero-vector ants). They were fed with sugar solution, marked to avoid repetition and tethered on a levelled air-cushioned trackball that was positioned ca. 30 cm above ground at 1.6 or 0.6 m away from the nest in the direction from which the ant had previously returned. Each ant was tested at each position in alternating order: if one ant was tested at 1.6 m first, the next ant was tested at 0.6 m first. The effect of order was tested and did not affect the results. Ants were filmed from above with a Panasonic Lumix DMC-FZ300 camera at 100 frames s^−1^ and their gaze directions (head orientation) were determined at 25 frames s^−1^ for 20 s at each location, starting 10 s after the start of recording. *x*/*y* positions were smoothed with a three-point averaging filter. The behaviour of ants at the two locations was assessed by determining and comparing the resulting vector length of circular gaze distributions, the circular standard deviation of the resulting vector direction and the resulting vector median directions. The distribution of variables at the two distances were statistically compared with two-sided Wilcoxon rank sum test.

In 2024, at the *M. impaternata* nest in the Mount Majura Nature Reserve (35°14.683′S, 149°10.282′E), the movements of returning foragers were recorded with a Sony FDR-AX100E 4K Camcorder in four situations: (1) on two consecutive days with the nest entrance and nest environment unobstructed (*n*=44), (2) on one day with the immediate nest environment covered by a rectangular sheet of Glad Wrap (Clorox Australia Pty Ltd; *n*=7), with a hole in the centre allowing access to the nest entrance, (3) on another day with the Glad Wrap replaced by a sheet of cotton cloth (*n*=8) and (4) on the fifth day with the nest entrance closed by a piece of Blu Tack (Bostik Australia Pty Ltd; *n*=12). Ant paths were digitized at 25 frames s^−1^ and from this I determined the time it took homing ants from 30 cm away from the nest to reach within 5 cm of the nest. Distributions were statistically compared using the Kruskal–Wallis test.

Finally, to assess the potential visual location information available from an ant's perspective, a Ricoh Theta S panorama camera was mounted sideways on the end of a drawer slide resting on the ground and pulled from a hideout to minimize artefacts with a string along 60 cm long transects at a height above ground between 2 and 5 cm. Video sequences were recorded at 30 frames s^−1^ in a north–south direction at two sites, one close to and the other directly at the Mount Majura nest site where the paths of five ants were recorded (35°14.50290′S, 149°10.09474′E). The video sequences were unwarped to rectangular panoramas (1920×960 pixels) with Ricoh Theta software, exported to image sequences with Vegas Pro 15.0 (Magix Software GmbH) and RMS image differences were calculated with a custom-written MATLAB program.

## RESULTS

Over the distances recorded, *Myrmecia* sp. foragers moved along generally nest-directed paths, but from their perspective, over complex terrain, with dense patches of vegetation alternating with few areas of bare ground (Fig. 1). The paths reflect frequent needs of detouring and of navigational corrections. Given that all five ants were returning from nearby trees and were intercepted without catching them, they may have partly been guided by path integration information, although *M. croslandi* have been shown in displacement experiments to ignore that information as long as there is guidance available from the landmark panorama (Narendra et al., 2013). In all five cases, ants changed their heading direction when they reached 0.5 to 1 m of the nest (examples indicated by arrowheads in Fig. 1).

**Fig. 2.**
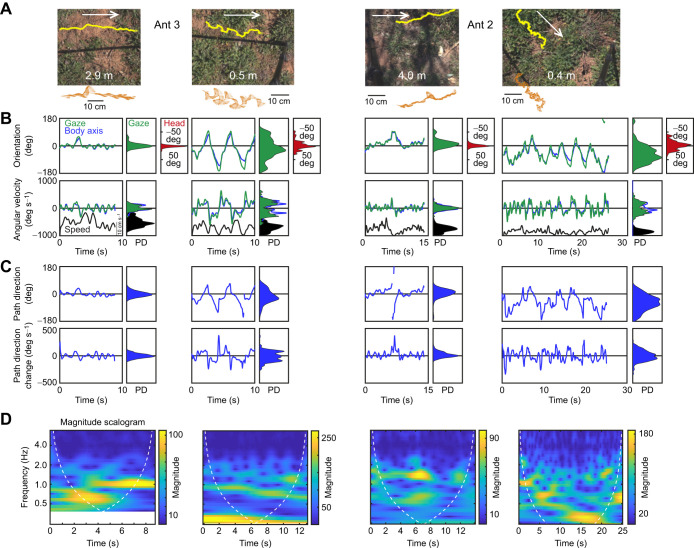
**The change in behaviour as ants approach the nest.** For two ants, examples of dynamic variables are shown comparing their behaviour at some distance from the nest and within 50 cm of the nest. (A) Path segments of the two ants at two distances from the nest as indicated. White arrows point into the direction of movement. Position and gaze directions (head orientation indicated by arrows) for the same sequences are shown below the camera images. (B) Top row: time series for the same sequences of gaze direction (green) and longitudinal body axis orientation (blue) together with probability densities (PD) on the right for gaze direction (green) and head orientation relative to longitudinal body axis orientation (red). Bottom row: same for angular velocities of the head (green), the angular velocity of the longitudinal body axis (blue) and the speed of locomotion (black). (C) Top row: time series of path direction (blue) and probability densities to the right. Bottom row: same for changes in path direction. (D) Magnitude scalograms of the angular velocity of the head as a result of a wavelet analysis using MATLAB's cwt function. Parts of A and B have been published in modified form in Zeil (2023).

**Fig. 3.**
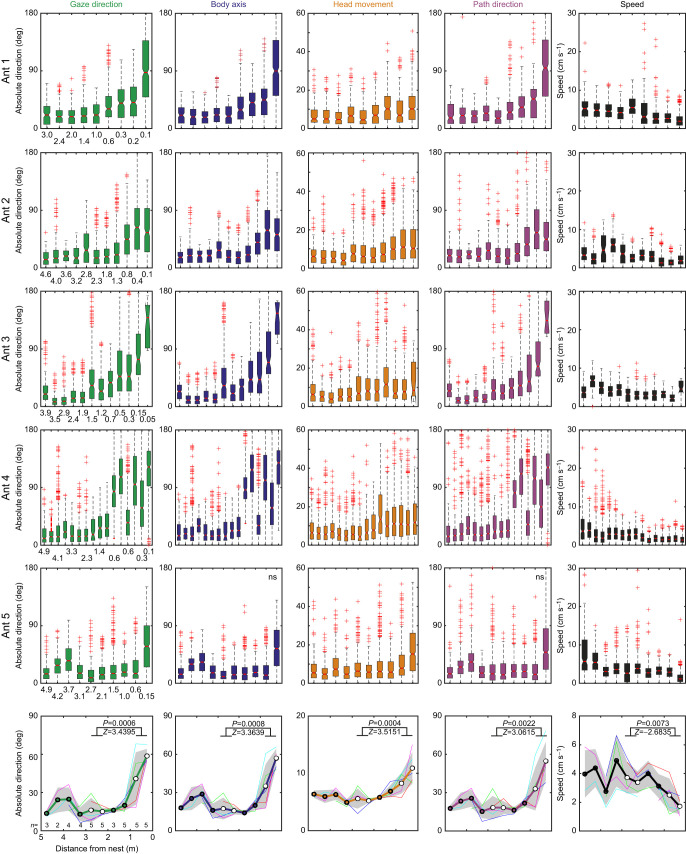
**The change in behaviour as ants approach the nest.** Box and whisker plots show absolute gaze direction (green), absolute orientation of the longitudinal body axis (blue), absolute head orientation relative to the longitudinal body axis (orange), absolute path direction (mauve) and speed (black) over distance from the nest for each recorded path segment of all five ants. Medians are indicated as horizontal red lines. Non-overlapping notches between boxes indicate that the true medians differ with 95% confidence. Bottom row: thin lines show for each ant medians of equal samples at 0.5 m distance bins. Thick lines and circles show the mean of medians and standard deviation indicated by the grey shaded area, with *n*-values at the bottom indicating the number of ants contributing. Statistical results are from a two-sided Wilcoxon rank sum test between medians at 2–3 m and 0–1 m distance bins (marked by white circles).

Within this range of the nest, the ants' behaviour changed quite dramatically in other ways. Two detailed examples show that the ants increased their scanning amplitudes (Fig. 2B) and their changes in heading direction (Fig. 2C) as they came closer to the nest location. They also made larger head movements and they slowed down. A wavelet analysis of the time course of gaze direction changes (green angular velocity traces in bottom panels of Fig. 2B) confirms that gaze oscillation frequency patterns changed depending on distance from the nest, with higher frequencies of ca. 1–1.5 Hz dominating at distance from the nest and gaze oscillations of frequencies below 0.5 Hz characterizing the ants' scanning behaviour close to the nest. As they approached the nest location, all five ants (Fig. 3) showed a consistent and statistically significant pattern of gradually larger scanning amplitudes (gaze direction), increased head movements, larger changes in heading or path direction and a decrease in walking speed (Fig. 3, bottom row).

**Fig. 4.**
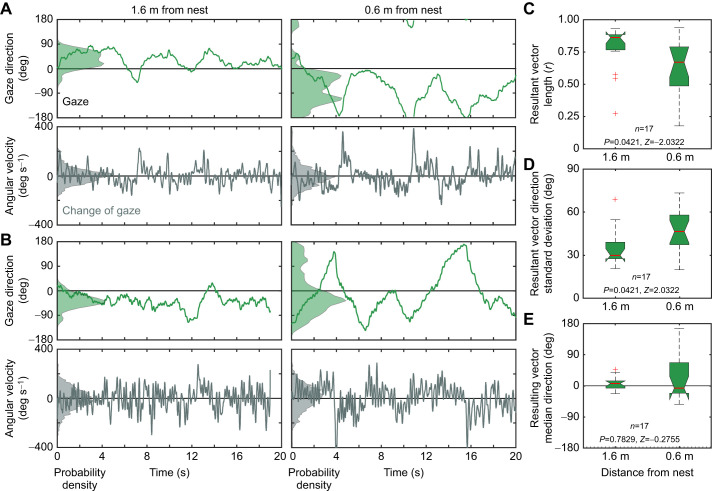
**The change of scanning dynamics of ants on a trackball.** (A,B) Detailed behaviour of two *M. croslandi* ants tethered on a trackball. Left column panels show the time course of gaze direction relative to nest direction at zero (light green) and the angular velocity of the head (dark green) when the trackball was positioned 1.6 m away from the nest; right column panels show the same when the trackball was positioned 0.6 m from the nest. Distributions on the left in each panel show the probability density for each variable. Gaze direction was recorded at 120 frames s^−1^ and smoothed with a 13-point (108 ms) running averaging filter. (C–E) Box and whisker plots comparing, for 17 *M. impaternata* ants, the resulting vector lengths (C), vector standard distributions (D) and vector median directions (E) at 1.6 and 0.6 m distance, together with two-sided Wilcoxon rank sum test statistics.

This conspicuous change in behaviour – at least as far as gaze direction is concerned – is elicited by views of the landmark panorama close to the nest and not by other cues on the ground such as tactile, olfactory or local visual cues, or the state of the path integrator that the ants may experience close to the nest: ants walking on a trackball 30 to 50 cm above ground showed the same switch in scanning behaviour when moved on a camera dolly from 1.6 to 0.6 m away from the nest location (Fig. 4A,B). At the larger distance from the nest, their gaze oscillated roughly around the goal direction, whereas close to the nest location their gaze swept at much larger amplitudes across the panorama (detailed examples in Fig. 4A,B). A large sample of ants tethered on a trackball at 1.6 and 0.6 m distance from the nest confirms this observation (Fig. 4C–E): the ants' gaze was more directed in the nest direction at 1.6 m, with the mean resultant vector length *r* being significantly larger at 1.6 m compared with 0.6 m (Fig. 4C) and the standard deviation of the resultant gaze vector direction being significantly smaller at 1.6 m compared with 0.6 m (Fig. 4D). The median direction of the resulting vector was close to zero at both distances, meaning that the ants' gaze is nest-directed in both situations (Fig. 4E).

**Fig. 5.**
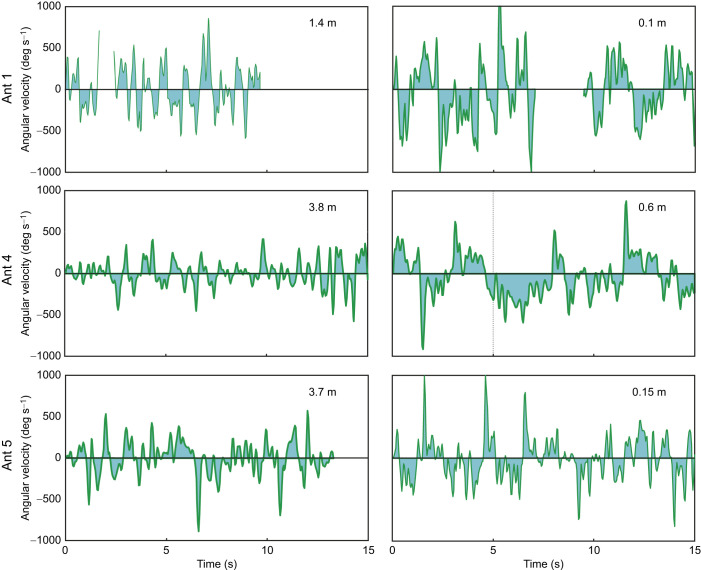
**The change of scanning dynamics as ants approach the nest.** Three examples (top to bottom rows) of the change in gaze direction at some distance from the nest (left column) and close to the nest (right column) of freely homing ants (see Fig. 1). Head angular velocity has been smoothed with a three-point running average filter. Note that close to the nest, ants reverse gaze direction less frequently.

Note that the change in scanning behaviour from 1.6 to 0.6 m distance is not so much driven by changes in angular velocities, but by a change in the pattern of alternating turns to the left and to the right (dark green traces in Figs 4A,B and 5). Close to the nest, the ants kept turning longer in one direction, before reversing direction, as has been described previously for ants on a trackball placed over the nest and along the foraging corridor 6.5 m away from the nest (Murray et al., 2020).

**Fig. 6.**
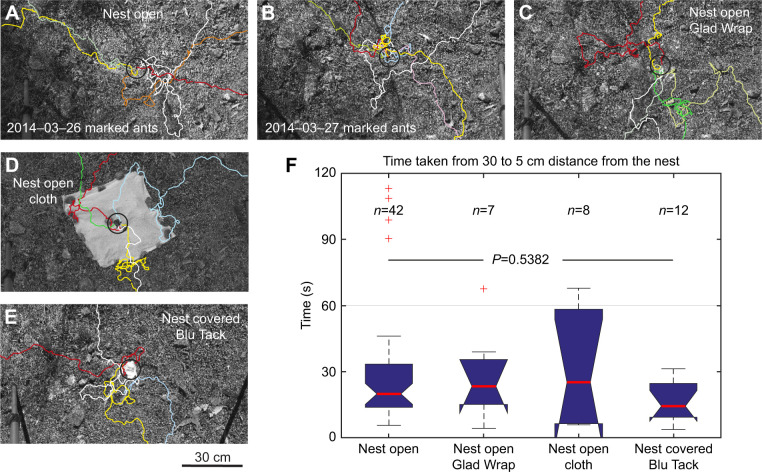
**Pinpointing the nest with local cues obstructed.** (A,B) Example paths of experienced *M. impaternata* foragers returning to the nest on two consecutive days. Ants had been marked as they participated in trackball experiments 20 days before these paths were recorded. (C) Example paths of ants returning to the nest environment covered by a thin sheet of transparent plastic (Glad Wrap). (D) Example paths of ants returning to the nest environment covered by a cotton cloth. (E) Example paths of ants returning to the nest that has been plugged with Blu Tack. A 10 cm diameter black circle is centred on the nest entrance. (F) Boxplots of the time taken by ants in the four conditions to reach the nest from a distance of 30 cm. *P*-value from a Kruskal–Wallis test, indicating that the four data sets come from the same distribution.

However, the change in scanning behaviour and the corrective manoeuvres ants perform as they approach the nest may also be due to their searching for, and being guided by, local visual, olfactory or tactile ‘landmarks’, including olfactory cues emanating from the nest entrance itself. To investigate the potential role of such cues in the ants' ability to pinpoint the nest entrance, I determined the time it took returning foragers to reach the nest entrance from a distance of 30 cm away under four conditions: (1) with the nest entrance and the local environment undisturbed (Fig. 6A,B), (2) with a sheet of thin, transparent plastic (Glad Wrap, Fig. 6C) or (3) of cotton cloth surrounding the open nest entrance (excluding local cues, Fig. 6D) and (4) with the nest entrance sealed with a Blu Tack plastic plug (excluding olfactory cues emanating from the nest itself, Fig. 6E). Although ants showed some initial reluctance to walk on the plastic and the cloth sheets, their time to reach to within 5 cm of the nest entrance did not differ between the four conditions (Fig. 6F).

**Fig. 7.**
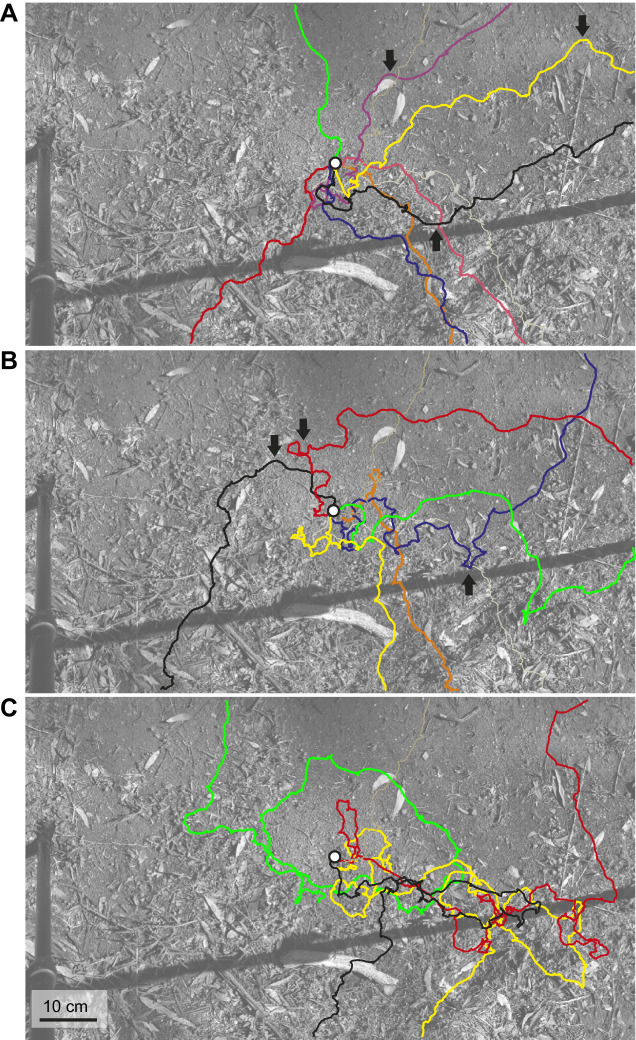
**The final approach paths of ants close to the nest.** Examples of final homing paths of *M. croslandi* ants sorted by eye into relatively direct approaches (A), approaches involving repeated changes in direction (B) and those involving extensive search (C). Arrows in A and B point to distinct corrections to path directions eventually leading to the nest entrance. Note also that ants enter the nest from all directions.

The paths shown in Fig. 6A,B are from experienced foragers returning to their undisturbed nest. The ants had been marked 20 days before these paths were recorded when they had participated in the trackball experiments (Fig. 4). Note the extended search some of these experienced foragers engage in before pinpointing the nest. Observing *Myrmecia* foragers returning to their undisturbed nest thus can be agonizing: although some ants approach the nest along fairly straight paths that potentially are guided by path integration information (Fig. 7A), more often than not, returning foragers can walk a few centimetres past the nest entrance before correcting their path (Fig. 7B) or spend significant amount of time searching (Fig. 7C). These search paths of *M. croslandi* foragers do not resemble the systematic search patterns observed in ant species that rely heavily on path integration (e.g. Schultheiss et al., 2015), which are characterized either by increasing loops centred on the goal location as defined by the path integration system (*Cataglyphis*; Wehner and Srinivasan, 1981), or by a search drifting in the home vector direction in ants that only partially follow the path integration vector (*Melophorus*; Narendra et al., 2008). A distinctive feature of the paths taken by returning *M. croslandi* foragers is the abrupt changes in heading direction at various distances from the nest entrance that realign the paths with the nest direction (examples indicated by black arrows in Fig. 7A,B). This feature, taken together with the previous observation that ants increase their scanning amplitude when approaching the nest, suggests that the ants are searching for information which drives the navigational decisions they make, rather than systematically covering an area, driven by the probability of the nest being there (e.g. Wehner and Srinivasan, 1981).

**Fig. 8.**
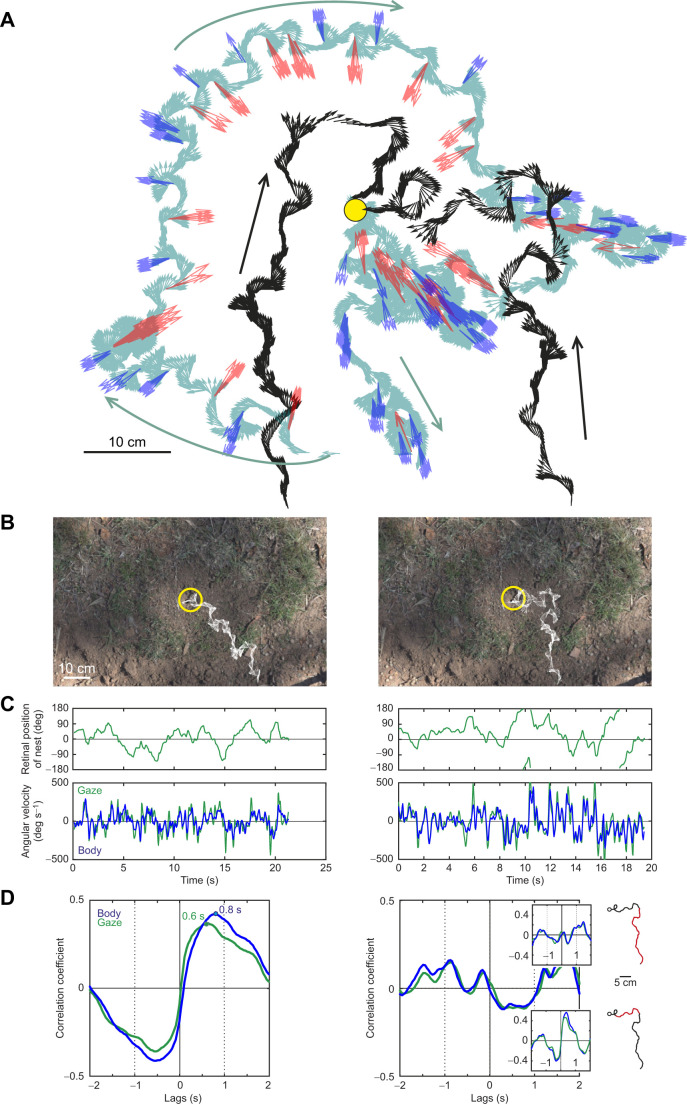
**Return path guidance I.** (A) Gaze directions (arrows) of *M. croslandi* ants during a typical learning walk (pale green) and during two examples of return walks (black). Learning walk and return walks not by the same ant. Instances during the learning walk when the ant looks into the nest direction are marked red and when she looks away from the nest blue. Nest marked by yellow circle. Learning walk modified from Zeil and Fleischmann (2019). (B) Two examples of gaze directions of two ants approaching the nest entrance (yellow circle) superimposed over the camera image. (C) Top row: for the paths shown in B, the nest direction in the visual field of the ants over time. Bottom row: same for the angular velocity of gaze (green) and of the longitudinal body axis (blue). (D) Cross-correlation between the nest direction and the angular velocities of gaze (green) and of the longitudinal body axis (blue). Peak correlation delays are marked by dots. Overall correlation for the path on the right does not show a distinct maximum, but the path shortly before and after a change in direction does. Relevant path segments are marked in red.

The learning walks *M. croslandi* perform when first exiting the nest and after a disturbance (Jayatilaka et al., 2018; Zeil and Fleischmann, 2019) provide a guide as to what information returning foragers may be looking for and which they may be accessing before choosing a heading direction that will bring them closer to the nest entrance. During learning walks, an example of which is shown in Fig. 8A, ants walk in loops around the nest and repeatedly turn to face the nest direction (indicated by red arrows in Fig. 8A) alternating with turns into the opposite direction (indicated by blue arrows in Fig. 8A). The ants thus have the opportunity to remember both attractive, nest-directed views and repellent views seen when pointing away from the nest (Jayatilaka et al., 2018; Murray et al., 2020). They may even remember any view and associate it with the nest direction, as supplied by the path integration system at any location during the learning walk (Müller and Wehner, 2010; Graham et al., 2010; Stürzl et al., 2016; Jayatilaka et al., 2018). If returning foragers are guided by such scene memories that are associated with nest direction, we would expect a delayed and positive correlation between the nest direction in the visual field of a returning ant and her turning rate: views associated with the nest to the left should lead to left turns and views associated with the nest to the right should lead to right turns, with the size of turns proportional to the difference between current gaze direction and the direction of the nest.

**Fig. 9.**
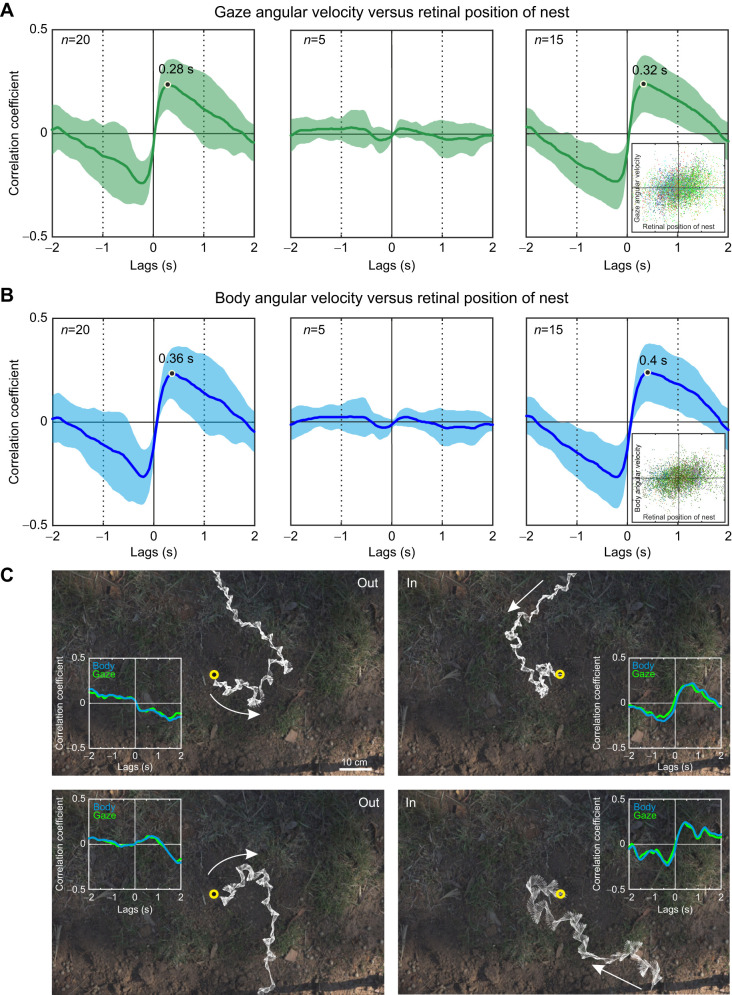
**Return path guidance II.** Average cross-correlation functions between gaze angular velocity (A) and body axis angular velocity (B) versus the nest direction in the visual field of homing *M. croslandi* ants. Thick lines show means and shaded areas standard deviations. Left column shows results for 20 return walks, centre column results for five walks with no pronounced modulation of the correlation function and right column results for the remaining 15 return walks. Delays at maximum are indicated in the panels on the left and on the right. Insets in the right panels show scatter plots of gaze and body angular velocity (−600 to 600 deg s^−1^) against retinal position of the nest (−180 to 180 deg) for 15 return walks at 0.32 and 0.4 s delay, respectively. (C) Delayed positive correlations between nest direction in the visual field of ants and head and body angular velocities are specific to in-coming paths. Shown are two examples of outgoing and incoming paths of two individually identified ants (top and bottom) together with the cross-correlation functions. Nest entrance is marked by the yellow circle.

This is indeed what I found. For two examples, the paths and gaze directions of returning foragers are shown in Fig. 8B as white arrows overlaid on the top-down camera image of the nest area. The time course of the retinal position of the nest (the difference between current gaze direction and the direction of the nest) and the angular velocities of head (gaze) and longitudinal body axis are shown in Fig. 8C. The cross-correlation functions between retinal position of the nest and the angular velocities of gaze and body have maxima at delays of 0.6 and 0.8 s, respectively, for the whole recorded path on the left (Fig. 8D). No such clear correlation exists for the path on the right, but this is due to the fact that there is no correlation during the initial approach (top inset, Fig. 8D right), whereas there is a strong correlation with shorter delays for the subsequent path before and after the change in path direction heading toward the nest (bottom inset, Fig. 8D right). The mean correlation functions for 20 return walks confirm the positive correlation between retinal position of the nest and both gaze and longitudinal body axis angular velocities (Fig. 9A,B). Delays vary between 0.28 and 0.32 s for gaze angular velocity and between 0.36 and 0.4 s for longitudinal body axis angular velocity depending on whether five examples of walks without clear modulation of the correlation function (middle panels, Fig. 9A,B) are excluded (compare left and right column panels in Fig. 9A,B). These correlations are continuous across retinal positions of the nest and are thus not only caused by ants responding when they face directly away from the nest (see scatter plot insets in the right panels of Fig. 9A,B). Moreover, the correlations are specific to incoming paths as illustrated by the outgoing and incoming paths of two individually identified ants in Fig. 9C.

**Fig. 10.**
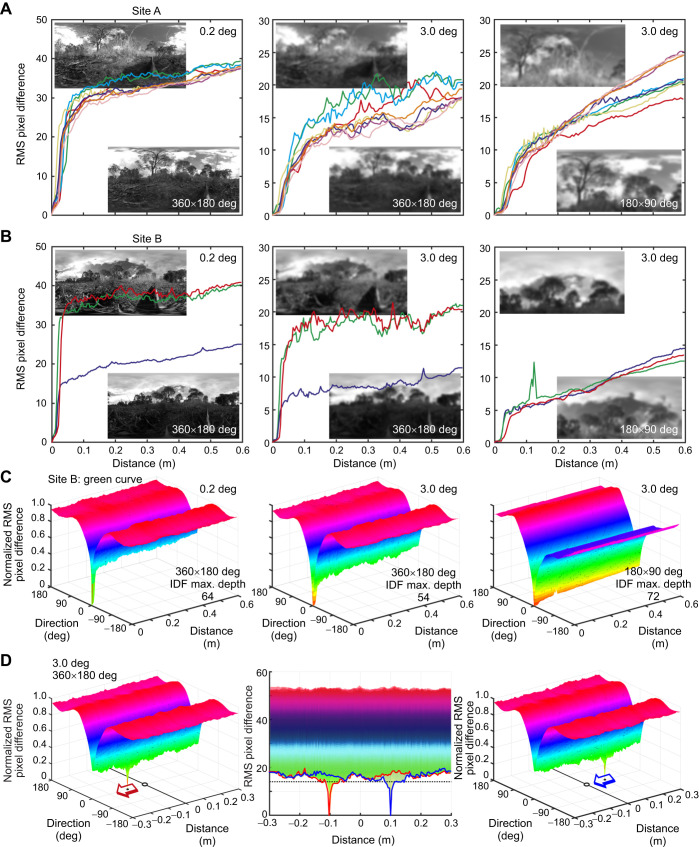
**The catchment areas of panoramic snapshots along 60 cm transects close to the ground.** Root mean square (RMS) pixel differences over distance from the reference image at zero distance (translational image difference functions, transIDF) are shown for (A) eight transects at different states of illumination at site A and (B) two transects when sunlit (red and green) and one transect when the sun was behind clouds (blue) at site B. Left panels show the transIDFs at 0.2 deg resolution, centre panels with the images low-pass filtered with a Gaussian with 3 deg full width at half maximum, both assuming a visual field of 360 deg horizontal and 180 deg vertical extent, and right panels assuming a visual field of 180 deg horizontal and 90 deg vertical extent (above the horizon). (C) The rotational image difference function (rotIDF) normalized to maximum over distance for the transect shown in green in B and for the same three conditions. Note that the transIDF traces the minimum of the rotIDF at zero degrees across distance and that the depth of the rotIDF is much larger compared with that of the transIDF. (D) The image difference functions for two reference images with the same orientation, but one (left) pointing away from an assumed nest location at distance zero (marked by a circle) and the other (right) pointing at the assumed nest. Reference image orientation is indicated by arrows. Centre panel shows both IDFs overlayed and collapsed along the direction dimension. The transIDFs of the right view (blue) and the left view (red) are emphasized by thick lines. Site A was close to and site B directly at the Mount Majura nest, where the paths of five ants were recorded (Fig. 1). Note that some of the ‘ruggedness’ of the transIDFs is due to jerky movements of the camera.

Taking these correlations as indicating that returning ants make navigational decisions depending on their recognition of similarities between memorized and currently experienced views, the question arises how much navigational guidance panoramic views can provide at such a very local scale. At a location close to trees (site A, Fig. 10A), global image differences due to translation (translational image difference function, transIDF) do indeed slowly decrease initially along a 60 cm path toward the reference location at zero distance and then sharply decline from approximately 10 cm to the goal, with some variation owing to changes in illumination (Fig. 10A, left panel). Importantly, the image difference gradient into the reference location widens when images are low-pass filtered with a Gaussian of 3 deg full width at half maximum (Fig. 10A, centre panel). Restricting the assumed visual field to a horizontal extent of 180 deg and to the dorsal part of the image (90 deg vertical extent, Fig. 10, right panel) smooths the transIDFs because ground clutter contributes less to image differences.

Further away from trees, at the location of the Mount Majura nest where the five ants were tracked home (Fig. 1), the catchment area of a reference snapshot, as defined by a descendible gradient (see Zeil et al. 2003; Stürzl et al., 2015; Zeil, 2023), is smaller, because distant visual features contribute less to the panoramic scene (Fig. 10B, site B). Again, restricting the visual field to the scene above the horizon leads to a smoothing of transIDFs (Fig. 10B, right panel). However, it is important to note that image differences owing to translation (Fig. 10A,B) are dwarfed by those generated by rotation (rotIDF, Fig. 10C). The values of the transIDF at the bottom edge of the IDF surface shown in Fig. 10C at direction zero (same as the green transect in Fig. 10B) are less than 30% of the depth of the rotIDF for most of the length of the transect. The directional information (rotIDF) provided by a reference image is thus much more robust or salient compared with the positional information (transIDF) (Fig. 10C). Counterintuitively, this poses a problem for visually homing ants approaching the nest.

I illustrate the issue in Fig. 10D. Imagine that an ant during her learning walk had memorized two views with the same orientation on opposite sides of the nest: one ‘attractive’ view pointing at the nest (Fig. 10D, right panel, blue arrow) and one ‘repellent’ view pointing away from the nest (Fig. 10D, left panel, red arrow). The IDFs of both snapshots are shown in the respective panels. Now imagine that the ant returns to the nest from the right and walks in the direction in which both views were memorized. Both views would be equally familiar to the ant up to the point approximately 15 cm from the nest where the IDF minimum of the attractive view (blue line in centre panel Fig. 10D) becomes smaller than that of the repellent view (red line in centre panel Fig. 10D). This similarity of closely spaced attractive and repellent views close to the goal may explain why homing ants display signs of uncertainty when they come close to the nest: they look around more, they change path direction more frequently and they slow down (e.g. Fig. 3), a behaviour that is also reproduced by a certain class of homing models (see Discussion).

## DISCUSSION

Returning foragers of *Myrmecia* spp. change their behaviour when coming close to their nest: they slow down, look around more and change their heading direction more frequently (see also Buehlmann et al., 2018). This change of behaviour contains elements of search and may signify uncertainty. It is also seen in ants walking 30–50 cm above ground on a trackball that is positioned 1.6 or 0.6 m away from the nest, indicating that this change in behaviour is largely driven by the visual scene changes and not by local olfactory, tactile or visual cues on the ground (see also Murray et al., 2020; Le Moël and Wystrach, 2020). In addition, experiments with different kinds of covers around and on the nest entrance show that ants can pinpoint the nest without the aid of such local cues, including olfactory cues emanating from the nest entrance itself. The detailed analysis of return paths within 60 cm or so of the nest revealed distinct corrections of heading direction eventually leading to the nest entrance, but also the fact that foragers can walk a few centimetres past the nest entrance before making final corrections. During most returns, the current misalignment between gaze direction and the direction to the nest is followed with mean delays between 0.28 and 0.4 s by both gaze and body turns that tend to align the ant with the nest direction.

It is thus tempting to conclude that pinpointing the nest by *Myrmecia* foragers is purely guided by global visual information, in particular, because their path corrections close to the nest are similar to those executed by homing wasps, which turn appropriately when encountering learning flight views associated with the nest being in the left or in the right visual field (Stürzl et al., 2016). Olfactory cues emanating from the nest entrance have been shown to be important guides in *Cataglyphis* ants (Steck et al., 2009, 2011; Steck, 2012; Buehlmann et al., 2012). However, they are unlikely to be involved in *Myrmecia* homing, considering that the ants can miss the nest entrance by centimetres, but can also pinpoint it approaching from all directions, when potential cues around the nest entrance are covered and when the nest entrance is closed (Fig. 6). Such manipulations have demonstrated that digger wasps, *Microbembex monodonta*, for instance, also rely exclusively on visual cues to pinpoint their hidden nest entrances with centimetre accuracy (Cormons and Zeil, 2023). However, the distinct corrective manoeuvres of ants approaching the nest could involve tactile (e.g. Seidl and Wehner, 2006; Merkle, 2009) or olfactory landmark cues (e.g. Steck et al., 2009, 2011; Steck, 2012; Buehlmann et al., 2015) that ants may have memorized during their learning walks associated with the direction of the nest entrance at the location at which they are encountered. It would actually be surprising if ants during their learning walks did not associate any detectable olfactory, tactile or visual change with the current direction to the nest. Yet, they clearly are able to pinpoint the nest without such potential cues.

The scanning behaviour of ants during their learning walks suggests that they systematically sample views around the nest, pointing toward and away from the nest from different compass directions (Fig. 8A; Jayatilaka et al., 2018; Zeil and Fleischmann, 2019). Considering that the nest entrance is visually inconspicuous, this sampling process must be guided by path integration (Müller and Wehner, 2010; Graham et al., 2010; Fleischmann et al., 2018) and in principle would allow ants to associate any view, irrespective of its orientation relative to the nest direction, with the home vector direction (Jayatilaka et al., 2018; Zeil, 2023). Alternatively, ants may learn attractive, nest-directed and repellent views when oriented away from the nest and use them in a push–pull fashion when homing. The changes of scanning behaviour by homing ants approaching the nest as described here and by Murray et al. (2020) can be explained by assuming that attractive and repellent view memories drive gaze and path oscillations (Murray et al., 2020; Le Moël and Wystrach, 2020): encounters with attractive, nest-directed views cause small oscillations and straighter paths, whereas encounters with repellent views cause large oscillations and larger changes in path direction.

Irrespective of the different ways in which attractive and repellent views may drive the behaviour of homing ants, the idea does not require views acquired around the nest to be in any way special or treated differently from those acquired along routes. But it does lead to the counterintuitive conclusion that for a homing ant, even without the contribution of path integration, the directional signal derived from views is much stronger at larger distances from the nest than close to it. The reason being that attractive views on the approach side of the nest are more similar to the current view, compared with repellent views on the opposite side of the nest (Narendra et al., 2013; Murray et al., 2020; Le Moël and Wystrach, 2020). Uncertainty increases close to the goal, because attractive, nest-directed views and repellent views directed away from the nest become more and more similar at opposite compass bearings. In this conceptual framework, there is thus no difference between route and nest views, but the range over which they provide information on nest location will depend on the distance distribution of visual features in any particular environment (Stürzl and Zeil, 2007). The particular distance distribution at any one site will also determine at what distance from the nest opponent views become too similar, leading to the change of behaviour as described here.

This is confirmed here by determining the catchment areas of panoramic views at a very fine spatial scale of centimetres around the nest, as close as possible to the viewpoint of ants (Fig. 10). Although global image differences on their own allow to pinpoint a location with centimetre accuracy, rotational image differences dominate to such a degree that opponent views can only be discriminated when an ant has come very close to where a view was recorded. Outside this small catchment area, opponent views will appear equally ‘familiar’ to the ant and, therefore, cannot provide directional guidance (Murray et al., 2020; Le Moël and Wystrach, 2020). It would seem that the only way for ants to solve this fundamental problem without local textural guidance is to scan, move and change direction until the exact location and orientation of a learned view is reached, can be distinguished from other views and therefore can direct the ant's next move. Importantly, this uncertainty exists, regardless of whether ants memorize any view associated with a home vector, or only those they experience when they are aligned in either direction parallel to the home vector, pointing toward or away from the nest. It is tempting to interpret the correlation between current misalignment with the nest direction and delayed corrective actions (Fig. 9) as evidence that ants indeed memorize all learning walk views tagged with the home vector direction. This possibility has been discussed previously (Jayatilaka et al., 2018; Zeil, 2023) and finds support though neural modelling (Wystrach, 2023 preprint). The correlation is relatively weak, owing to the random elements of search triggered by the similarity of views with the same orientation close to the nest.

In conclusion, experimental results showed that *Myrmecia* ants pinpoint their nest entrance using information derived from global image comparisons, not from local visual, tactile or olfactory cues. As they approach their nest, they show signs of uncertainty, indicated by increased scanning and path direction changes (see Wystrach et al., 2014). This change in behaviour cannot be explained by the decreasing reliability of path integration information close to the goal (e.g. Hoinville and Wehner, 2018), because zero vector ants also show this behavioural change close to the nest. An analysis of the navigational information provided by attractive and repellent local views, as they may be memorized during learning walks, suggests that the uncertainty ants experience close to the goal is a consequence of the similarity of closely spaced views. Modelling of homing paths using natural scenes and different assumptions on what ants learn during learning walks is required to test this conclusion. But it would also be interesting to see whether this uncertainty and the ants' response to it can be manipulated by the introduction of large, visually salient landmarks close to the nest.
